# Analysis on epidemiological characters and HIV care continuum of HIV-infected students: a retrospective cohort study in Shandong province, China

**DOI:** 10.1186/s12879-023-08476-6

**Published:** 2023-07-27

**Authors:** Xingguang Yang, Ling Li, Na Zhang, Lianzheng Hao, Xiaoyan Zhu, Haiying Yu, Guoyong Wang, Dianmin Kang

**Affiliations:** Shandong Center for Disease Control and Prevention, Jinan, 250014 China

**Keywords:** Acquired immune deficiency syndrome, Human immunodeficiency virus, Young student, Continuum of care, Antiretroviral therapy

## Abstract

**Backgroud:**

The proportion of HIV-infected students in China showed an increasing trend. This study aimed to identify the epidemiological characteristics and the HIV care continuum for HIV-infected students in Shandong Province, China.

**Methods:**

Case report and follow-up data of HIV-infected students were obtained from the National HIV/AIDS comprehensive response information management system. Logistic regression analyses were used to analyze the associating factors of HIV-infected students accepting CD4 + T cells (CD4) test and antiviral therapy (ART) in 30 days, and ArcGIS software was used for the spatial anlysis.

**Results:**

From 2017 to 2019, 403 HIV-infected students were reported in Shandong Province. The majority of them were male (99.5%) and transmitted through homosexual sexual activity(92.1%). Most of them lived in Jinan city and Qingdao city. 68.5% (276 cases) accepted CD4 test in 30 days, and 48.6% (196 cases) started ART in 30 days. The heterosexual transmitted cases (AOR = 0.458, 95%CI: 0.210–0.998), patients accepting HIV care in western area (AOR = 0.266,95%CI: 0.147–0.481) were less likely to test CD4 within 30 days; patients aged 23–25 (AOR = 2.316, 95%CI: 1.009–5.316) and patients who had tested CD4 within 30 days (AOR = 4.377; 95%CI: 2.572–7.447) prefered to receive ART within 30 days; patients accepted HIV care in central area (AOR = 0.407; 95%CI: 0.251–0.657) and western area (AOR = 0.508; 95%CI: 0.261–0.989) and patients diagnosed by voluntary blood donation (AOR = 0.352; 95%CI: 0.144–0.864) were less willing to receive ART in 30 days.

**Conclusions:**

The HIV care continuum of HIV-infected students in Shandong Province still needed strenghthing. More health education and case management should be done for cases transmitted through heterosexual behavior, accepted HIV care in central and western area, and diagnosed by voluntary blood donation.

## Introduction

In 2020, there was a daily increase of 4000 new human immunodeficiency virus (HIV) infections globally, 31% of which were adolescents aged 15–24 years, defined as the youth by the United Nations [1]. In adolescents aged 10–24 years, Acquired immune deficiency syndrome (AIDS) was among the top 10 causes of Disability Adjusted Life Years [2]. In China, during 15–24 years adolescents, the proportion of students was 44.4% and the crude reporting rate of HIV/AIDS among 15–24 years students was rising approximately 4.2 times from 1.24 to 100,000 persons in 2010 to 5.19 per 100,000 persons in 2019 [3, 4].

Shandong province is located on the east coast of China, the Yellow River mouth area, and is one of the provinces with the most developed economy and the strongest economic strength in China [5]. Shandong province is also famous for hometown of Confucius. People here place a high priority on education, so there are a huge numbers of students and people very concerned about the spread of HIV among students [5]. 15–24 years students were in sexually active phase, could skillfully use Internet and social software to find friends, proned to high-risk sexual behaviors [6]. One research about the high-risk behaviors of people newly diagnosed with HIV/AIDS showed that high income and high educational level were risk factors for high-risk behaviors [7]. In Shandong province, the use of drugs is common among young MSM with higher education [8]. Drug abuse would promote unprotected sex [9] and group sex [10]. All these above lead to a great risk of HIV transmission among students in Shandong province [11].

Because sexual transmission is the main infected route of HIV for students [3], so promoting condom use would be very effective in reducing transmission [12]. However, studies have shown that condom use rate is not high among MSM population in Shandong Province due to pleasure, fluke and the other reasons [13]. High-quality HIV care is another useful way for achieving targets of ending HIV epidemic [14–16]. Accept ART after diagnosis as soon as posssible can prevent opportunistic infections and achieve viral suppression [17, 18], prolong the survival time of patients [19, 20]. Furthermore, ART can reduce viral load of HIV-infected individuals and therefore reduce sexual transmission [21–23]. The updated Chinese guidelines for diagnosis and treatment of HIV/AIDS (2018) recommend early initiation of ART for HIV prevention. Linkage to HIV care were also effective intervetion methods. CD4 test was an important part of links-to-care interventions [24, 25].The level of CD4 cells of patients can reflect the degree of disease progression [26, 27], the immune status and treatment effect of patients [14], consequently provided the evidences for therapeutic regimen adjustment for doctors as a common indicator. Timely and regularly detecting CD4 could reduce the occurrence of drug resistance [28], achieve viral suppression, sequentially reduce transmission.

Zhu XY et al [29] conducted a study on the timeliness of ART for HIV infected individuals in Shandong Province, which showed that young people aged 15–24 were less likely to accept ART timely. However, there has not study on HIV care status of HIV-infected students and not research finding the factors of student cases accepting HIV care up to now.

This study analyzed the epidemiological characters and HIV care status of HIV-infected students in Shandong province during 2017 to 2019, and explored the associating factors of student cases accepting CD4 test and ART during 30 days after diagnosis. Based on these findings, we could provide effective prevention and control measures to make HIV-infected students accecpt HIV care continuum as soon as possible, consequently avoiding HIV transmission among students in Shandong Province.

## Materials and methods

### Data collection

Case report data and follow-up data of HIV/AIDS among students from 2017 to 2020 were obtained from the National HIV/AIDS comprehensive response information management system, excluding Hong Kong, Macao, Taiwan and foreign cases. The system was established by Chinese Center for Disease Control and Prevention in 1985 and available online since 2005 [30]. Our analysis did not include those who was diagnosed during 2020 to allow all cases having 24 months following to get information about HIV care after diagnosis.

HIV-infected students in this study were defined as HIV-positive individuals who were students, aged 15–24 years and diagnosed by physicians at the medical institutions in China based on national standards [31–33]. Blood samples were initially screened for HIV by enzyme-linked immunosorbent assay (ELISA). Positive results were confirmed by western blot. Data on the sociodemographic characteristics, disease status, routes of transmission, and risky behaviors were collected by the staff of the local Center for Disease Control and Prevention (CDC) using standardized forms. All data used in this study were anonymized to protect the privacy of participants. The research protocol was approved by the Ethical Committee of Prevention Medicine of Shandong Center for Disease Control and Prevention (ethical code: 2021-20).

### Defintion

According to geographical and economic conditions, we divided Shandong province into four areas, eastern area (including Qingdao City, Dongying City, Weifang City, Rizhao City, Yantai City and Weihai City), central area (Jinan City, Tai ‘an City, Zibo City, Linyi City) and western area (Liaocheng City, Dezhou City, Binzhou City, Heze City, Jining City, Zaozhuang City) [17].

### Statistical analysis

The statistical analysis was performed by the SPSS Statistical Package for Social Sciences (version 21.0; SPSS Inc., Chicago, IL, USA). Linear-by-Linear Association (LLA) test was used to analyze the temporal trend over these years [34]. Univariate Logistic regression analyses were conducted to estimate the potential risk factors for HIV infected students accepting HIV care. Variables with statistical significance in univariate analysis and other variables considered to be influential by other studies were included in binary Logistic regression analysis. A *p-value* < 0.05 were considered significant. Adjusted odds ratios (AOR) and 95% Confidence Intervals (CI) for HIV infected students receiving HIV care were reported for each explanatory variable in the final models.

The spatial distribution of HIV-infected students and the proportion of they receiving HIV care were mapped using ArcGIS software (version 10.2; ESRI Inc, Redlands, CA, USA), at the county level of Shandong Province.

## Results

### Socio-Demographic and behavioural characteristics of HIV infected students

**Fig. 1:**
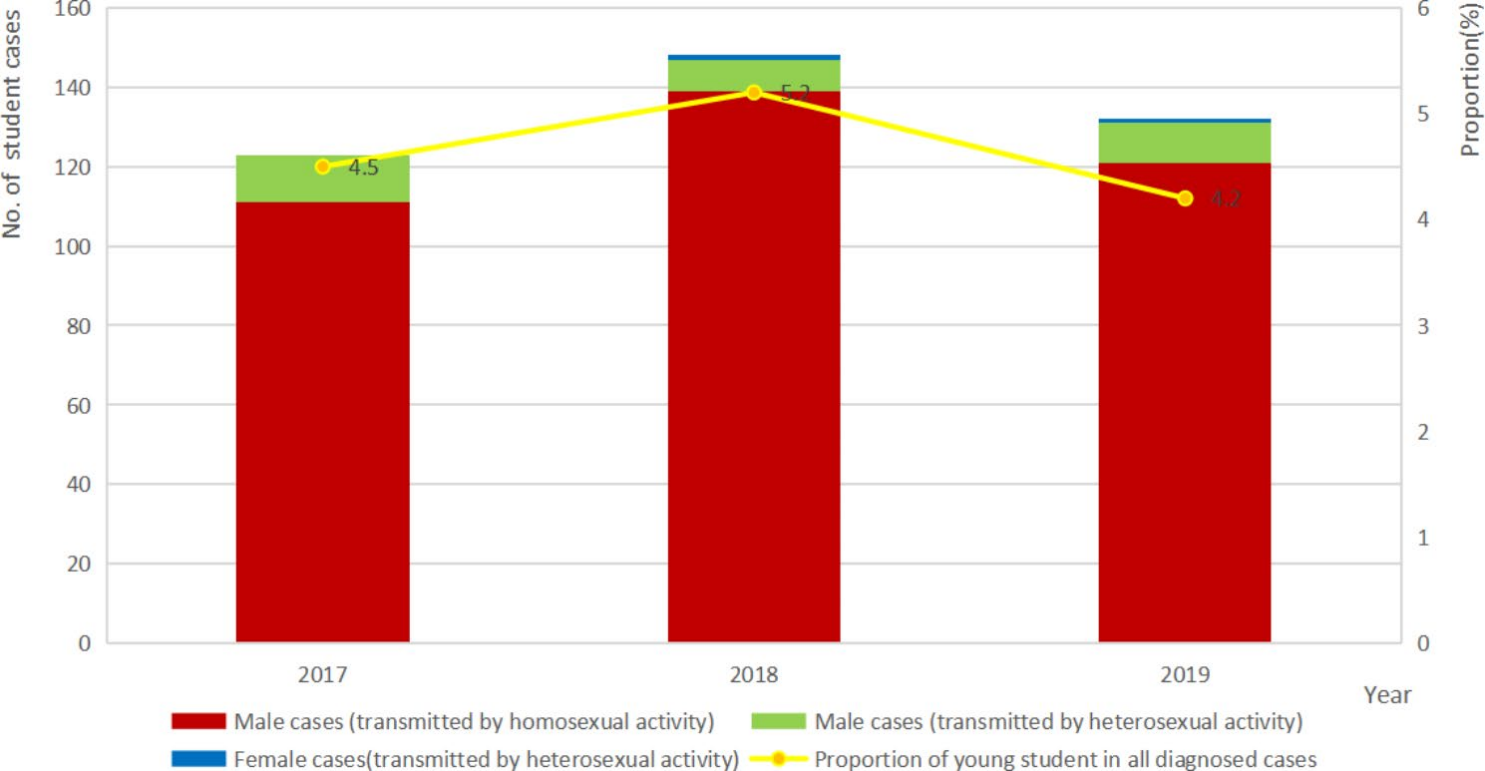
The temporal trend and the characteristics of HIV infected students, 2017–2019

There were 403 cases of HIV infected students in Shandong Province, China during 2017–2019, with 123, 148, and 132 cases in the respective years. During 403 student cases, 401 cases were male (99.5%), 329 cases were in 18–22 age group (81.6%), 178 cases received HIV care in eastern area (44.2%), 343 cases had college degree or above (96.5%). All these cases were sexually transmitted, of which 92.1% were homosexual transmitted and 7.9% were heterosexual transmitted. All CDCs in shandong province have an HIV voluntary counseling and testing (VCT) clinic providing free test. Blood banks and detention centers also offer free test. People also could pay for a test in hospital when they have a surgery, blood transfusion, or other physical discomfort. In our research, VCT (199 cases, 49.4%) and hospitals (141 cases, 35.0%) were the main resources for HIV diagnosis. The proportion of students cases among all HIV infected individuals diagnosed in Shandong during 2017–2019 were 4.5%, 5.2%, and 4.2% respectively, and no significant change was observed (LLA statistic = 0.323, p > 0.05), as shown in the Fig. 1.

### The status of HIV care among HIV-infected students

**Fig. 2:**
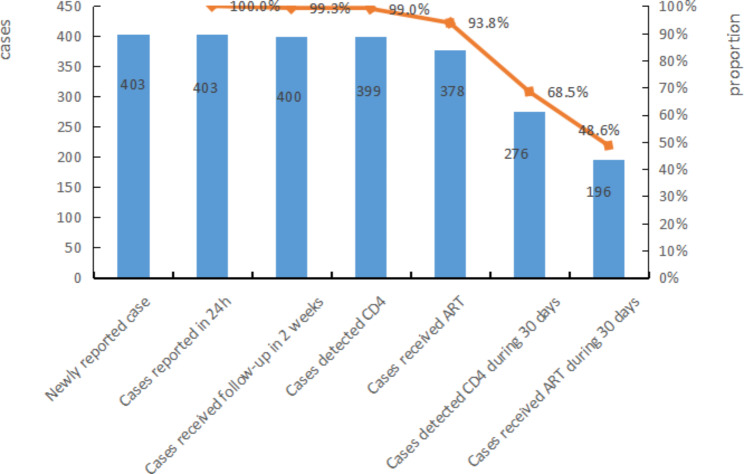
The status of HIV-infected students aceived HIV care

Of the 403 HIV infected students, all cases were reported to the National Data Information System within 24 h, 400 cases (99.3%) received the first follow-up from the local CDC within 2 weeks, 399 cases (99.0%) were tested for CD4, and 378 cases (93.8%) received ART, 276 cases (68.5%) tested CD4 within 30 days after diagnosis, and 196 cases (48.6%) received ART within 30 days after diagnosis, as shown in the Fig. 2.

### The associating factors of HIV infected students receiving CD4 test in 30 days

From 2017 to 2019, the proportion of HIV infected students received CD4 test during 30 days was 67.5%, 67.6% and 70.5%, respectively. There was no significant increase or decline over the study period (LLA statistic = 0.267, P > 0.05).

Univariate analysis showed that HIV infected students received CD4 test were related with the route of transmission and the district they accepting HIV care. Variables with statistical significance in univariate analysis and other variables considered to be influential by other studies were included in binary Logistic regression analysis, and the results showed that the heterosexual transmitted cases were less likely to detecte CD4 within 30 days than homosexual transmitted cases (AOR = 0.458, 95%CI: 0.210–0.998), patients accepting HIV care in western area were less likely to detecte CD4 within 30 days than those in eastern area (AOR = 0.266,95%CI: 0.147–0.481), as shown in Table 1.

**Table 1:** The associating factors of HIV infected students receiving CD4 test

Variables	Student cases (n,%)	Cases receiving CD4 test in 30 days (%)	Univariate analysis		Multivariate analysis	
			OR(95%CI)	P	AOR(95%CI)	P
**Age of diagnosis**						
15–22	372 (92.3)	254 (68.3)				
23–25	31 (7.7)	22 (71.0)	1.136(0.507–2.542)	0.757	0.864 (0.371–2.010)	0.734
**Degree of education**						
high school or less	60 (14.9)	38 (63.3)				
College degree or above	343 (85.1)	238 (69.4)	1.312(0.740–2.328)	0.353	1.080 (0.586–1.991)	0.804
**Areas of HIV care**						
Eastern area	178 (44.2)	136 (76.4)				
Central area	158 (39.2)	109 (69.0)	0.687 (0.424–1.114)	0.128	0.663 (0.404–1.089)	0.104
Western area*	67 (16.6)	31 (46.3)	0.266 (0.147–0.481)	0.000	0.286 (0.155–0.528)	0.000
**Route of transmission**						
Homosexual	371 (92.1)	261 (70.4)				
Heterosexual*	32 (7.9)	15 (46.9)	0.372 (0.179–0.771)	0.008	0.458 (0.210–0.998)	0.049
**Resource of diagnosis**						
VCT	199 (49.4)	142 (70.6)				
Hospital	141 (35.0)	90 (64.7)	0.763 (0.481–1.211)	0.251	0.876 (0.536–1.431)	0.598
Voluntary blood donation institution	33 (8.2)	22 (66.7)	0.831 (0.379–1.822)	0.644	0.856 (0.372–1.967)	0.714
The other	30 (7.4)	22 (73.3)	1.143 (0.481–2.712)	0.762	1.090 (0.433–2.744)	0.856
**Baseline CD4 count**						
≤ 500/ul	281 (69.7)	193 (68.7)				
>500/ul	122 (30.3)	83 (68.0)	0.970 (0.615–1.532)	0.897	0.988 (0.612–1.595)	0.961

Notes: * means P < 0.05, the difference is statistically significant.The proportion of cases that received CD4 test within 30 days was determined by dividing the student PLWHs who received CD4 test within 30 days by all the reported student PLWHs.

### The associating factors of HIV infected students receiving ART in 30 days

From 2017 to 2019, the proportion of student patients received ART during 30 days increased from 26.8% to 2017 to 54.5% in 2019 over the study period (LLA statistic = 11.958, P < 0.05).

Univariate analysis showed that patients receiving ART within 30 days were associated with age, areas of HIV care, resource of diagnosis and received CD4 test within 30 days or not. Binary Logistic regression analysis showed, student cases aged 23–25 prefered to receive ART within 30 days than that aged 15–22 (AOR = 2.316, 95%CI: 1.009–5.316); patients accepted HIV care in central area (AOR = 0.407; 95%CI: 0.251–0.657) and western area (AOR = 0.508; 95%CI: 0.261–0.989) were less willing to receive ART within 30 days than those in eastern area; patients diagnosed by voluntary blood donation were less willing to receive ART in 30 days than those detected by VCT (AOR = 0.352; 95%CI: 0.144–0.864); patients who tested CD4 within 30 days were more likely to start ART in 30 days (AOR = 4.377; 95%CI: 2.572–7.447), as shown in Table 2.

**Table 2:** The associating factors of HIV infected students receiving ART

Variables	Student cases (n,%)	Cases receiving ART in 30 days (n,%)	Univariate analysis		Multivariate analysis	
			OR(95%CI)	P	AOR(95%CI)	P
**Age of diagnosis**						
15–22	372 (92.3)	148 (39.8)				
23–25*	31 (7.7)	19 (61.3)	2.396 (1.130–5.083)	0.023	2.316 (1.009–5.316)	0.048
**Degree of education**						
High school or less	60 (14.9)	20 (33.3)				
College degree or above	343 (85.1)	147 (42.9)	1.500 (0.842–2.673)	0.169	1.483 (0.788–2.794)	0.222
**Areas of HIV care**						
Eastern area	178 (44.2)	95 (53.4)				
Central area*	158 (39.2)	52 (32.9)	0.429 (0.275–0.668)	0.000	0.407 (0.251–0.657)	0.000
Western area*	67 (16.6)	20 (29.9)	0.372 (0.204–0.678)	0.001	0.508 (0.261–0.989)	0.046
**Route of transmission**						
Homosexual	371 (92.1)	157 (42.3)				
Heterosexual	32 (7.9)	10 (31.3)	0.620 (0.285–1.345)	0.226	0.862 (0.360–2.065)	0.739
**Resource of diagnosis**						
VCT	199 (49.4)	89 (44.3)				
Hospital	141 (35.0)	60 (43.2)	0.956 (0.618–1.478)	0.839	1.016 (0.625–1.652)	0.949
Voluntary blood donation institution*	33 (8.2)	8 (24.2)	0.403 (0.173–0.936)	0.035	0.352 (0.144–0.864)	0.023
The other	30 (7.4)	10 (33.3)	0.629 (0.280–1.412)	0.261	0.439 (0.178–1.079)	0.073
**Baseline CD4 count**						
≤ 500/ul	281 (69.7)	114 (40.6)				
>500/ul	122 (30.3)	53 (43.4)	1.125 (0.732–1.730)	0.591	1.129 (0.702–1.817)	0.616
**Received CD4 test in 30 days**						
No	127 (31.5)	25 (19.7)				
Yes*	276 (68.5)	142 (51.4)	4.324 (2.630–7.107)	0.000	4.377 (2.572–7.447)	0.000

Notes: * means P < 0.05, the difference is statistically significant. The proportion of cases that received ART within 30 days was determined by dividing the student PLWHs who received ART within 30 days by all the reported student PLWHs.

### Geographical distribution of HIV-infected students

**Fig. 3:**
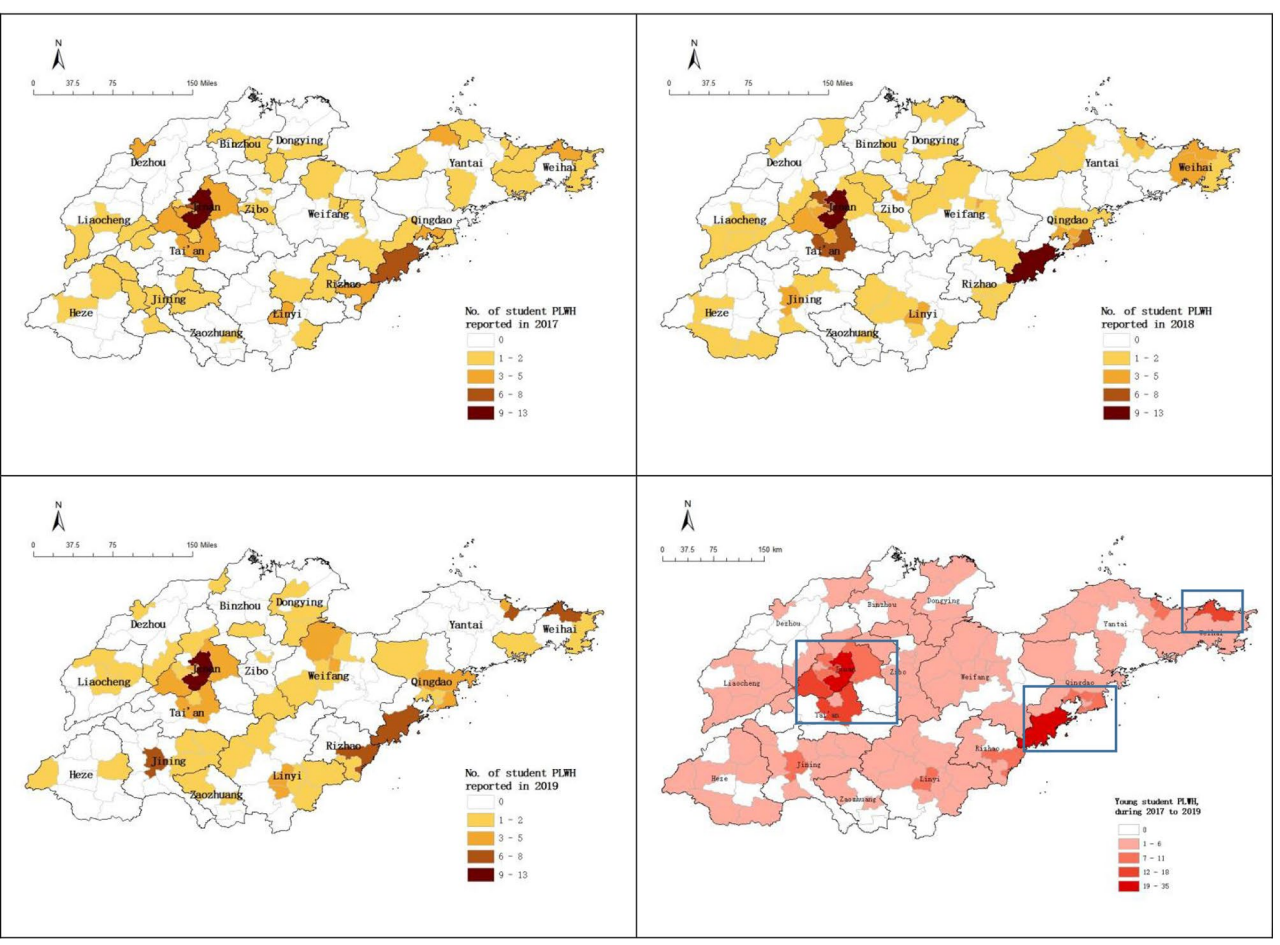
The annually spatial distribution of HIV infected students

Overall, 96 counties (96/139, 69.1%) reported student cases during 2017–2019. 55 counties (39.6%), 61 counties (43.9%), and 55 counties (39.6%) reported HIV infected students respectively in 2017, 2018, and 2019. The counties reporting cumulatively more than 13 cases were Licheng, Huangdao, Lixia, Daiyue, Huancui and Changqing district. Licheng, Lixia and Changqing, above these three districts belong to Jinan city (the capital of Shandong province), Huangdao district belongs to Qingdao city, Huancui district belongs to Weihai city, and Daiyue district belongs to Tai ‘an city, just like the blue box shown in Fig. 3.

**Fig. 4:**
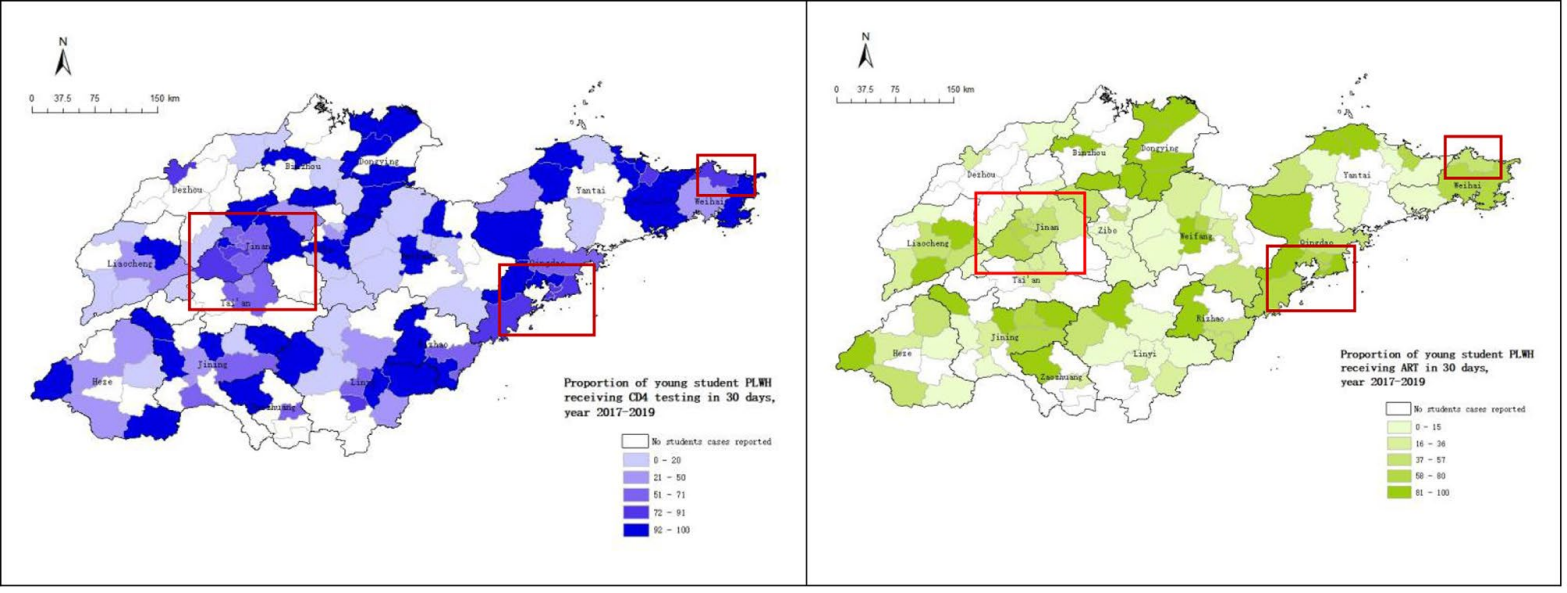
The geographical distribution of HIV infected student receiving CD4 test and ART in 30 days

In terms of geographical distribution, the proportion of cases receiving CD4 test and ART within 30 days in southeast coastal area showed higher than that in the inland area. In cities reported more cases, such as Jinan, Tai ‘an, Qingdao and Weihai in blue box (Fig. 3), the proportions of cases receiving CD4 test and ART within 30 days were not highest (just like the Fig. 4 showed). Meanwhile, cities with higher propotion of student cases receiving CD4 test and areas with higher propotion of receiving ART during 30 days were not consistent, as shown in Fig. 4.

## Discussion

This study analyzed the epidemiological characters and HIV care status of HIV-infected students in Shandong province during 2017 to 2019. From 2017 to 2019, 403 HIV-infected students were reported in Shandong Province, most of them were male and homosexual transmitted. They mainly distributed in Jinan city and Qingdao city. 276 cases (68.5%.) accepted CD4 test in 30 days, and 196 cases (48.6%) started ART in 30 days.The heterosexual route, area they accepted HIV care were associated with the timeliness of CD4 test. The age, accepted CD4 test within 30 days or not, diagnosis source and area they accepted HIV care were associated with the timeliness of ART.

This study shows that most student cases were detected through VCT, which may be related with that young students knew more AIDS related knowledge, they could actively look for professional help after unsafe sexual behavior. In Shandong Province, MSM occupied 92.1% of all the students cases, so publicity and education for MSM is the key for eliminating the spread of AIDS among young students. High-risk behaviors during them such as unprotected sex, multiple sex partners and temporary partner [35–37] still occurred due to the separation of knowledge, belief and practice. And this group is harder to reach for CDC staffs, some people reached the populations through collaboration with gay-friendly CBO [38].Non-occupational postexposure prophylaxis (nPEP) was an effective means of prevention [39], but the cost was high for students. Treatment and care interventions are still effective interventions for these populations.

In this study, 276 cases (68.5%) tested CD4 within 30 days after diagnosis; 196 cases (48.6%) received ARTwithin 30 days after diagnosis. Although these proportion were higher than the survey results of 15–24 years HIV infected students in Hangzhou city conducted by Yan et al [40], they were still not high. More efforts should be done to encourage young students to receive CD4 test and ART as soon as possible. Some studies found that delaying in CD4 test was associated with older age, low education level, injection or routes of transmission, HIV diagnosis in a hospital or in a detention center, and being a migrant worker [41]. However, in our study, young students had relatively similiar education level and age, so only the transmission route was found be associated with delaying in CD4 test.

A cross-sectional survey including HIV infected patient in 19 hospitals across Spain showed that people living with HIV (PLWH) didn’t start ART was associated with limited doctors and medical resource [42]. There was also research showed that medical staffs had influence on the ART effect of patient [43]. In Shandong Province, east coastal areas, including Qingdao, Weihai and Yantai city, et al., have more developed economy, abundant medical resources, and more higher level medical staff equipped to do HIV care. So this study showed that patients accepted HIV care in central area (AOR = 0.407; 95%CI: 0.251–0.657) and western area (AOR = 0.508; 95%CI: 0.261–0.989) were less willing to receive ART within 30 days than that in eastern area. We also found that cases aged 23 years and above prefered to receive ART within 30 days than those aged 15–22 (AOR = 2.316, 95%CI: 1.009–5.316), maybe attributing to students aged 15–22 were inconvenient to get and take medicine. 15–22 years old students mainly lived in school with their classes and did not want other people know their infectious status due to shame and stigma [44], all these will add difficulty for young students take medicine. Jiao et al [45] showed that digital intervention could improve ART adherence. Designing more privacy-friendly pill boxes for residential students may be beneficial for students to take medicine. Notably, this study found that patients diagnosed through voluntary blood donation were less willing to receive ART in 30 days than those detected through VCT (AOR = 0.352; 95%CI: 0.144–0.864). This may be related to a lapse of patients in referral to a treatment hospital from detection sites. Staffs in blood donation agency usually did not understand the treatment knowledge and the initiation time of treatment, so they could not adequately inform and refer patients. Study in Vietnam [46] also showed that clinical characteristics (time and facility at first time received HIV-positive result) were associated with HAART non-adherence. Liu et al [37] thoght people tried to know their infection status through voluntary blood donation maybe due to the stigma, so reduction of homosexuality- related stigma [37, 47] could be part of effective intervention efforts. We found that patients tested CD4 within 30 days were more likely to start ART in 30 days (OR = 4.377; 95%CI: 2.572–7.447). Patients detected CD4 early tend to have good compliance and high cooperation with medical personnel. Through subsequent follow-up and detection process, the interaction between medical personnel and patients was enhanced, so as student cases would more likely to cooperate to ART. Some studies [46, 48] showed that demographic (education levels) was associated with HAART non-adherence. However, this study does not found similar results, may be due to we chosed 15–24 years students as study object, whose demographic characteristics, such as education levels were similar.

The geographical distribution figure showed, about 20% of counties/districts in Shandong Province had not reported any young student cases during 2017 to 2019, and the reported cases were concentrated in economically developed cities such as Jinan, Qingdao, Yantai, Weihai. These places also have more colleges and universities. Chen et al [49] studied on the spatial distribution of student cases in Zhejiang Province also found similar characters. Some spatial analysis of HIV/AIDS also found that cases tend to be cluster in urban areas and areas with railways and roads [50]. The proportion of the young student cases receiving CD4 test and ART within 30 days in southeast coastal area showed higher than northwest inland area. But areas reported more student cases, such as Jinan, Tai ‘an, Qingdao and Weihai in the blue box, the rates of student cases received CD4 test and ART within 30 days were not higher. About geographical distribution, the proportion of student cases received CD4 test during 30 days was not consistent with student cases received ART. Maybe the distribution of medical resources is not completely reasonable for student cases. More targeted research is needed to understand the reasons of mismatches.

There are some shortcomings in this study. For example, although the data of the basic information system of AIDS prevention and control in this study can fully reflect the situation of young student PLWH, its epidemic characteristics may be affected by the intensity of detection. In addition, we only found the distribution of medical resources is not completely matching for student cases, but we haven’t found the reason in depth, more researches were needed in future.

## Conclusions

In conclusion, the HIV care of HIV infected students in Shandong Province still needed to be improved. Publicity, education and case management should be done for cases of heterosexual transmission, accepted HIV care in central and western areas, and detected through voluntary blood donation.

## Data Availability

The datasets used and analyzed during the current study are available from the corresponding author (Guoyong Wang, Email: yshhl@163.com) on reasonable request.

## Abbreviations

CD4: CD4 + T cells
ART: Antiviral therapy
HIV: Human immunodeficiency virus
AIDS: Acquired immune deficiency syndrome
ELISA: Enzyme-linked immunosorbent assay
CDC: Center for disease control and prevention
AOR: Adjusted odds ratios
CI: Confidence Intervals
VCT: Voluntary counseling and testing
nPEP: Non-occupational postexposure prophylaxis
